# The Role of Neuroglobin in Retinal Hemodynamics and Metabolism: A Real-Time Study

**DOI:** 10.1167/tvst.11.7.2

**Published:** 2022-07-08

**Authors:** Pardis Kaynezhad, Glen Jeffery, James Bainbridge, Sobha Sivaprasad, Ilias Tachtsidis, Anders Hay-Schmidt, Ranjan Rajendram

**Affiliations:** 1 Institute of Ophthalmology, University College London, London, UK; 2 Medical Physics and Biomedical Engineering, University College London, London, UK; 3 Department of Odontology, Panum Institute, University of Copenhagen, Copenhagen, Denmark

**Keywords:** neuroglobin, oxygen, retina, near-infrared spectroscopy, hemoglobin, mitochondria

## Abstract

**Purpose:**

In this study, we used broadband near-infrared spectroscopy, a non-invasive optical technique, to investigate in real time the possible role of neuroglobin in retinal hemodynamics and metabolism.

**Methods:**

Retinae of 12 C57 mice (seven young and five old) and seven young neuroglobin knockouts (Ngb-KOs) were exposed to light from a low-power halogen source, and the back-reflected light was used to calculate changes in the concentration of oxygenated hemoglobin (HbO_2_), deoxygenated hemoglobin (HHb), and oxidized cytochrome c oxidase (oxCCO).

**Results:**

The degree of change in the near-infrared spectroscopy signals associated with HHb, HbO_2_, and oxCCO was significantly greater in young C57 mice compared to the old C57 mice (*P* < 0.05) and the Ngb-KO model (*P* < 0.005).

**Conclusions:**

Our results reveal a possible role of Ngb in regulating retinal function, as its absence in the retinae of a knockout mouse model led to suppressed signals that are associated with hemodynamics and oxidative metabolism.

**Translational Relevance:**

Near-infrared spectroscopy enabled the non-invasive detection of characteristic signals that differentiate between the retina of a neuroglobin knockout mouse model and that of a wild-type model. Further work is needed to evaluate the source of the signal differences and how these differences relate to the presence or absence of neuroglobin in the ganglion, bipolar, or amacrine cells of the retina.

## Introduction

Neuroglobin (Ngb) is a neuron-specific, oxygen-binding globin protein that was discovered in 2000.^1^^,^^2^ Ngb concentration has been reported to be 100 times greater in the retina than in the brain, which suggests that an important function for Ngb would exist within the retina and that the Ngb knockout (Ngb-KO) would be the optimal model to study the role of this globin in the retina. The concentration of Ngb in the retina is ∼50- to 100-fold greater than that found in the brain, and its distribution strongly correlates with the localization of mitochondria and with relative oxygen demand.^3^^,^^4^ The function of Ngb is not yet fully understood, but different studies suggest its critical role in mitochondrial oxidative metabolism and neuroprotection, acting as an oxygen reservoir and a sensor to detect cellular oxygen concentration in order to protect neurons against hypoxia and oxidative stress.^5^^,^^6^

Neuroglobin is able to interact with reactive oxygen species and nitric dioxide and is able to avert cytochrome c–induced apoptosis.^6^ Seventy percent of Ngb is found in the mitochondrial compartment of the cell (neuron), and Ngb knockdown in primary ganglion cell cultures leads to compromised cell survival.^5^

Ngb expression has been measured in vitro in human and murine models; however, in vivo studies are highly valuable in the pursuit of gaining a better understanding of its mechanism and function. Interest in the possible roles of Ngb in the retina was renewed after a study in 2012 revealed Ngb immunoreactivity in some ganglion, bipolar, and amacrine cells in the inner nuclear layers through the use of a highly validated Ngb antibody and an Ngb-null mouse.^7^

Here, we examined Ngb-deficient and control young and old C57 Bl mice using broadband near-infrared spectroscopy (bNIRS) as a non-invasive optical technique to further understand the role of Ngb in normal retinal hemodynamics and metabolism.

bNIRS scans over 100 to 120 wavelengths at 1-nm resolution in the near-infrared region were used to measure relative changes in the tissue concentration of oxygenated hemoglobin (HbO_2_) and deoxygenated hemoglobin (HHb) and to provide information on mitochondrial oxidative metabolism by measuring changes in oxidized cytochrome c oxidase (oxCCO) with higher accuracy compared to conventional two- to three-wavelength NIRS systems.^8^ In this study, we used a previously described miniature bNIRS system, miniCYRIL,^9^^,^^10^ to investigate and compare normal retinal function in C57 mice, as well as in a Ngb-KO model.

## Methods

Ngb-deficient mice were generated as previously described.^7^^,^^11^ They had a genomic deletion of Ngb exons 2 and 3, resulting in a lack of Ngb protein. The mice were used with University College London ethics approval and under a UK Home Office project license (PPL 70/8379). All procedures conformed to the United Kingdom Animal License Act (1986), local regulations, and the ARVO Statement for the Use of Animals in Ophthalmic and Vision Research.

### Near-Infrared Spectroscopy

Seven young C57 mice (6–7 months old) and five old C57 mice (22 months old), as well as seven young Ngb-KO mice were anesthetized with 0.3 mL of a mixture of 6% ketamine and 10% Domitor (National Veterinary Services, Stoke-on-Trent, UK) and 84% H_2_O at 5 µm/g. The pupils were dilated with 1% tropicamide (Bausch & Lomb, Montpellier, France), and the cornea was lubricated with Viscotears (Novartis, Basel, Switzerland). Mice were unrestrained and placed on their side for real-time measurement of retinal oxygenation and hemodynamics, as well as metabolism, embedded in light within the 780- to 900-nm range using the miniCYRIL.

**Figure 1.**
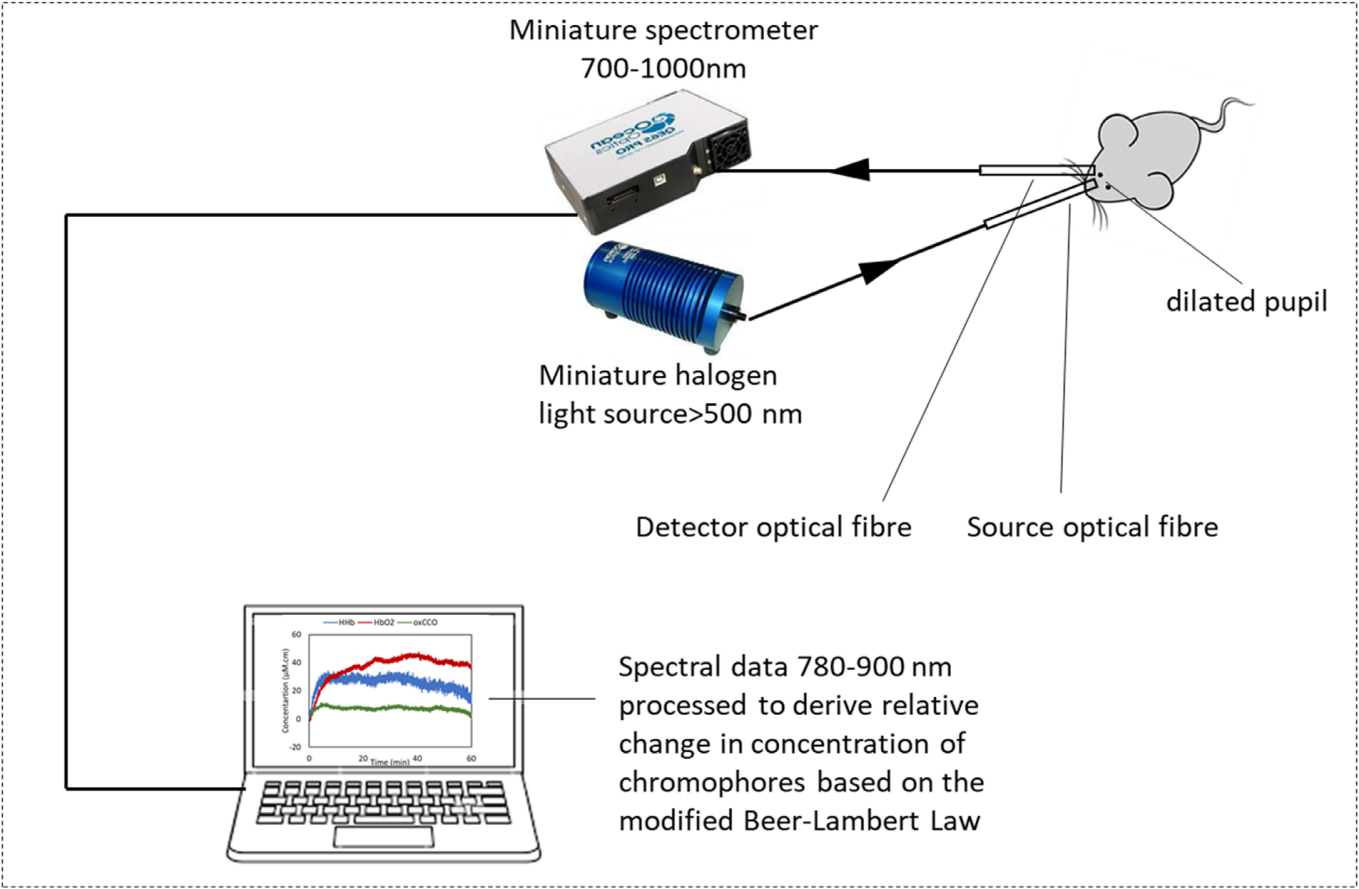
Schematic of the broadband NIRS experimental setup. Light (>500 nm) was delivered to the mouse's dilated pupil through an optical fiber. The reflected light (700–1000 nm) was collected via an identical optical fiber from the surface of the eye and delivered to a miniature spectrometer. The spectral data were transferred to a laptop, and real-time changes in the concentration of HbO_2_, HHb, and oxCCO were calculated every second from the relative changes in the attenuation of light between 780 and 900 nm.

A schematic of the experimental set up is presented in Figure 1. Experiments were performed in a darkened room (total irradiance, ∼ 2 × 10^−4^ W/m^2^), and a white light source (HL-2000; Ocean Optics, Dunedin, FL) was used to illuminate the retina with a yellow filter to remove the short wavelengths (cut-off, ∼<500 nm). Two custom-made optical fibers (engionic Fiber Optics, Berlin, Germany) were placed in front of the eye, mounted on stereotaxic probe holders at ∼40° to each other and perpendicular to the cornea to avoid specular reflection. The first fiber transmitted light from the source to the retina, and the second collected the back-reflected light and transmitted it to the spectrometer carrying within it metrics of oxygenation and metabolism (HbO_2_, HHb, and oxCCO), which were quantified using the UCLn algorithm based on a modified Beer–Lambert law equation^12^ described in Equation 1:

(1) $$\;\;\;\Delta \;\;\;C=\;\;\;\Delta \;\;\;A\left(\lambda \right)\cdot \;\;\;ɛ\;\;\;\left(\;\;\;\lambda \;\;\;\right)\cdot pathlength\left(\;\;\;\lambda \;\;\;\right)$$

UCLn is a least-squares regression analysis, which finds the best fit of chromophore concentration change Δ*C*, using the chromophore extinction coefficient, ε(λ); the measured change in attenuation, Δ*A*(λ), over 120-nm intervals covering 780 to 900 nm; and the optical pathlength of light through the tissue at each wavelength (λ).^13^

Here, the optical pathlength is different from the axial length of the eye, as the NIR light (780–900 nm) goes through multiple scatterings which adds to the pathlength. Also, the actual penetration depth is unknown, and computational modeling is required to estimate the optical pathlength; hence, all of the measurements of concentration are expressed in µM × cm. The old C57 mice and KO mice had clear visual axes, and no lens clouding was observed at the end of each experiment.

Experiments were performed and analyzed in MATLAB 2018b (MathWorks, Natick, MA). Spectral data from the retinae of mice were collected every second, and real-time changes in HHb-, HbO_2_-, and oxCCO-associated signals were recorded for 1 hour. The range of change in concentration of these chromophores was calculated over 1 hour, and the groups were compared using non-parametric Wilcoxon rank-sum tests.

### Histology

The retinae from perfusion phosphate-buffered saline (PBS) buffered, formalin-fixed Ngb-KO and WT mice were halved and cryopreserved in 30% sucrose in PBS for 5 days, when they were sectioned on a freezing stage microtome in four series of 4-µm sections and mounted on chrome-gelatin–coated object slides.

### Immunostaining

Sections were immunostained with a rabbit anti-Ngb antibody (made in-house, #4836; characterized by Hundahl et al.^11^); the antibody was diluted 1:30,000 in PBS plus 1% human serum albumin (hSA) plus 0.1% sodium azide and 0.1% Triton X-100 (hSA-PTA). Sections were rinsed in PBS for 10 minutes and incubated in 1% H_2_O_2_ in PBS for 5 minutes. Sections were rinsed three times in PBS for 10 minutes and preincubated in hSA-PTA for 1 hour before the primary antibody was added. Sections were incubated in primary antibody (i.e., rabbit anti-Ngb) overnight at 4°C and then rinsed three times in hSA-PTA for 10 minutes before being incubated with the secondary F(ab′)_2_ donkey anti-rabbit antibody (#711-066-152; Jackson ImmunoResearch Laboratories, West Grove, PA) diluted 1:2500 in hSA-PTA for 1 hour at room temperature. Sections were then washed three times in hSA-PTA and incubated for 1 hour at room temperature in an avidin–biotin complex (VECTASTAIN Elite ABC HRP Kit; Vector Labs, Burlingame, CA) diluted 1:50 in PBS. The sections were then rinsed three times in PBS and incubated for 15 minutes in 0.05% diaminobenzidine (Sigma-Aldrich, St. Louis, MO) plus 0.0001% H_2_O_2_. The chromogene reaction was stopped by washing 5 minutes in PBS plus 1% H_2_O_2_ followed by rinsing in Milli-Q water (MilliporeSigma, Burlington, MA). Hematoxylin was used for counterstaining (see Fig. 3).

## Results

### Near-Infrared Spectroscopy

**Figure 2.**
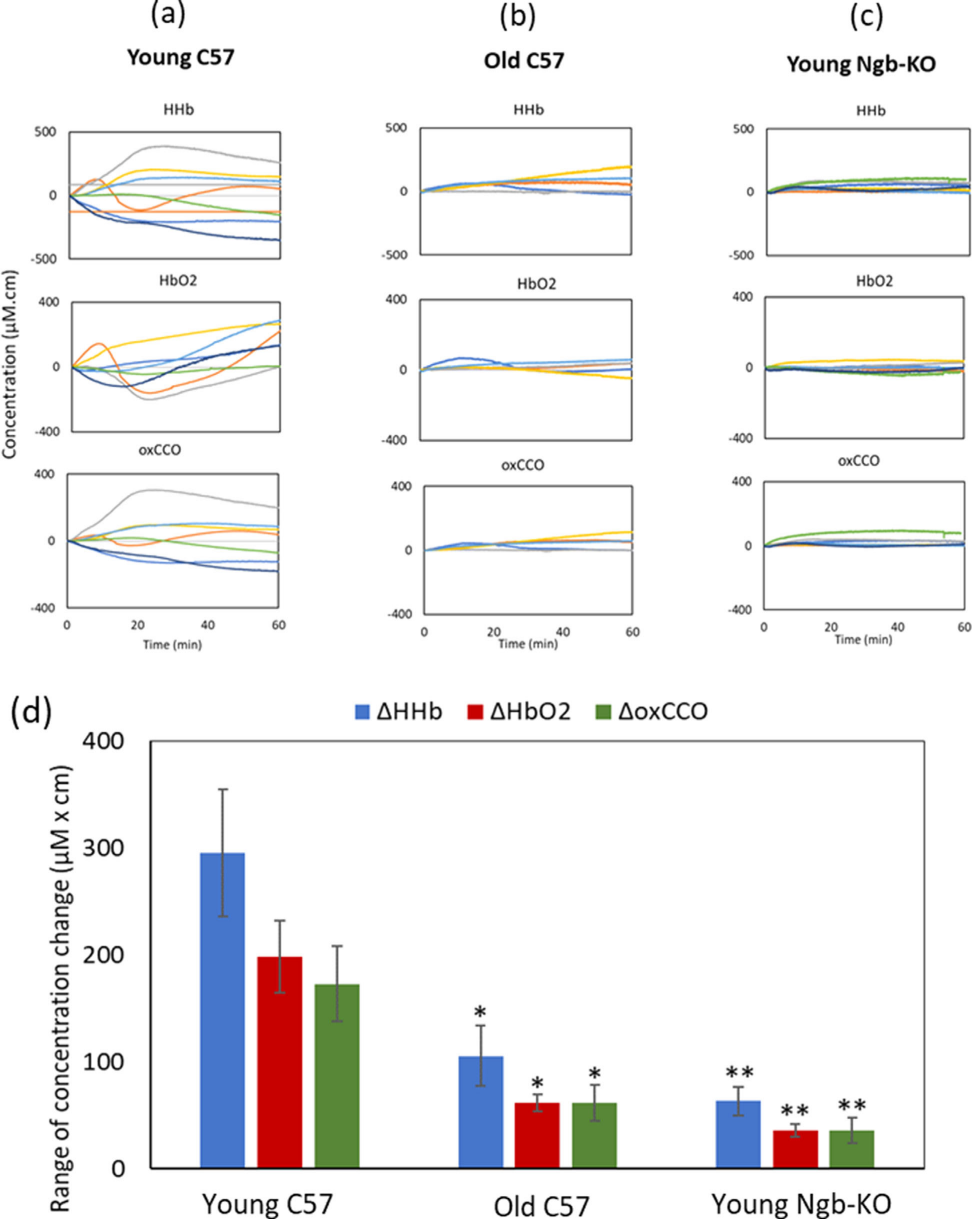
The role of Ngb in retinal hemodynamics and metabolism. (a, top to bottom) Baseline changes in retinal signals for HHb, HbO_2_, and oxCCO in young C57 mice (*n* = 7). (b, top to bottom) Changes in HHb, HbO_2_, and oxCCO in old C57 mice (*n* = 5). (c, top to bottom) Changes in HHb, HbO_2_, and oxCCO and young Ngb-KOs (*n* = 7). The greatest variation in all of the signals was observed in the young C57 mice; the signals were notably suppressed in the old mice and the Ngb-KOs. (d) Group data showing the range of signal change for concentrations of HHb, HbO_2_, and oxCCO during the measurement period (1 hour). The range of signal change for each chromophore is compared against the corresponding value in the young C57 group using non-parametric Wilcoxon rank-sum tests. *P < 0.05; **P < 0.005. *Error bars* are standard errors of the mean.

Baseline measurements of young and old C57 mice and young Ngb-KOs are presented in Figures 2a, 2b, and 2c, respectively. The signals show relative changes in HHb, HbO_2_, and oxCCO in the retinae of mice. The magnitude of signal change for all three markers for retinal hemodynamics and metabolism in the young C57 mice is remarkably greater compared to both the old C57 mice and the Ngb-KO mice. The mice in each group included both sexes; however, as expected, there were no sex differences in the metrics measured.

Group data also confirmed the significantly greater range of change (variability) in the signal, which can be attributed to the concentrations of HbO_2_, HHb, and oxCCO over an hour.

### Neuroglobin Immunostaining of the Retina

**Figure 3.**
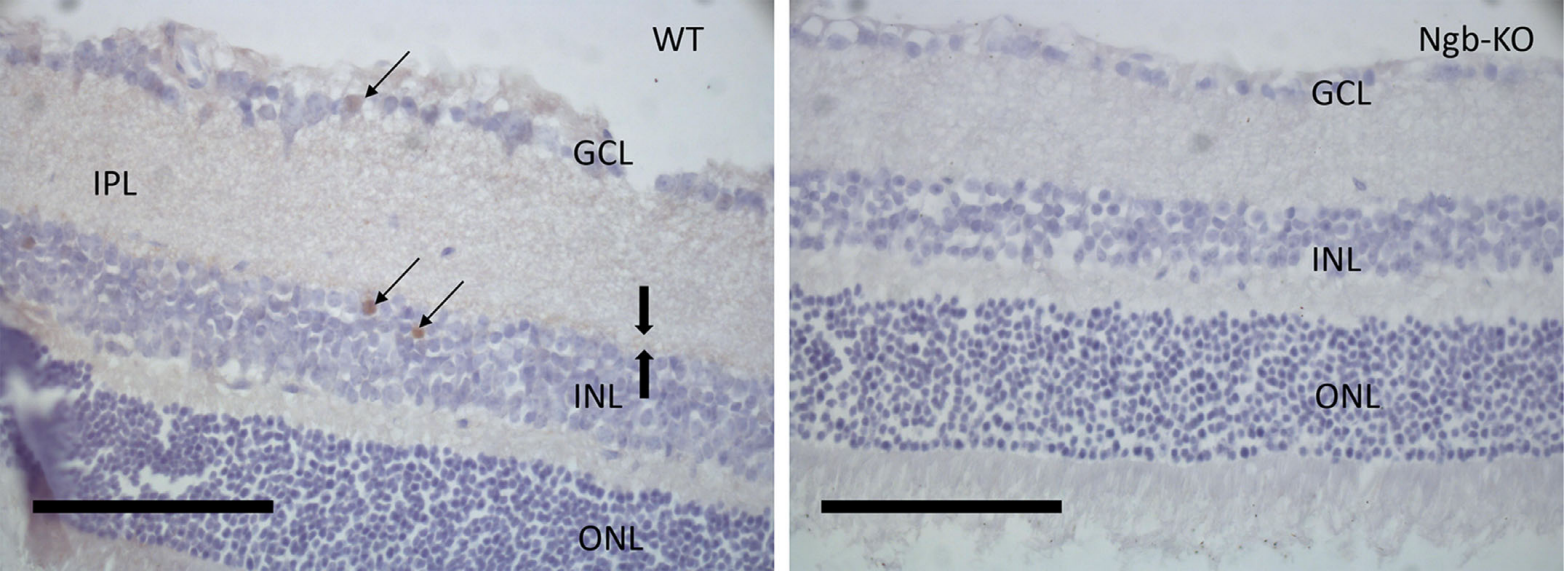
Retina sections were obtained and immunostaining was carried out as described in the Methods section. *Scale bar*: 100 µm. (a) Wild-type (WT) mouse retina section. Ngb-immunoreactivity (IR) nerve fiber staining (*bold* *arrows*), Ngb-IR cell body staining in GCL (*arrows*), and INL (*arrows*). (b) Ngb-KO mouse retina section. INL, inner nuclear layer; ONL, outer nuclear layer; GCL, ganglion cell layer.

The expression of Ngb in the wild-type mouse retinae was sparse and restricted to two layers. Strong Ngb immunoreactivity was seen in both perikarya and processes of a subpopulation of neurons in the ganglion cell layer and in amacrine cells of the inner nuclear layer (Fig. 3a). No Ngb immunostaining was seen in the Ngb-KO mouse retina (Fig. 3b). The antibody and its use have been previously validated.^7^

## Discussion

In this study, we examined neuroglobin-deficient (null) and control C57 Bl mice to assess real-time retinal oxygenation and metabolism using broadband NIRS in Ngb-KOs, as well as aged mice compared with young mice. The range of signal changes for concentrations of HHb, HbO_2_, and oxCCO was significantly reduced in the aged and the Ngb-KO models. The difference in the range of signal changes between young C57 mice and other groups became statistically significant from the first 20 minutes of the measurement; at 1 hour, the Ngb-KO mice had the lowest range of change in their blood and mitochondrial signals. These results are consistent with Ngb playing a role in modulating oxygen homeostasis, perhaps in response to differing metabolic demands of the retina.

During the measurement, the retina was continuously illuminated by low-power light (>500 nm), which revealed notably large variations in the signals for the concentration of HbO_2_, HHb, and oxCCO in young C57 mice. This could be due to the high expression of neuroglobin acting as an oxygen sensor in the ganglion cell layer and inner nuclear cell layer,^14^^,^^15^ continuously detecting oxygen concentrations at the cellular level and adjusting the supply to the retinal neurons accordingly.

The old C57 mice showed significantly suppressed variability in their retinal hemodynamics (changes in HHb and HbO_2_ NIRS signals) and oxidative metabolism (changes in oxCCO) in contrast to the young mice, which could be due to the decreased Ngb expression in the retinae of old mice, the same way that it is shown to be reduced in the aged rodent brain.^16^ Likewise, the Ngb-KOs showed the smallest variation in their retinal hemodynamics and metabolism during the measurement, which could be due to the lack of Ngb to regulate oxygen metabolism.

Although the results we present are clear, there is much that remains unknown. We cannot exclude the possibility that differences in the range of relative concentration signals are not the result of differences in the spectroscopic features associated with age and/or the absence of Ngb as a NIRS absorber in the retina. These may affect light scattering and absorption leading to varied pathlength and are determining parameters in the intensity and features of the back-reflected light from which the relative concentration of absorbers are quantified. We are not aware of any difference in neuroglobin distribution associated with the sex of the mice nor of any differences related to the NIRS technique described here. Regardless of the underlying mechanism behind the different hemodynamics and oxidative metabolism signals between normal and Ngb-KO mice, our results suggest that Ngb plays a role in retinal metabolism and, as such, potentially also retinal function. Although our technology does not provide a direct measurement of neuroglobin, the range of change in blood and mitochondrial signals (HHb, HbO_2_, and oxCCO) could provide a reliable real-time marker to investigate the way Ngb governs and regulates retinal oxygenation and metabolism in vivo. Therefore, our methodology may be a useful real-time technique to further study Ngb function in healthy normal aging and in disease. Further work will be needed to evaluate the source of the signal differences and how this relates to the presence or absence of neuroglobin in the ganglion, bipolar, or amacrine cells of the retina.
